# Sequential Increase in Complement Factor I, iC3b, and Cells Expressing CD11b or CD14 in Cutaneous Vasculitis

**DOI:** 10.1155/2022/3888734

**Published:** 2022-06-14

**Authors:** Dina Rahkola, Tiina Lipitsä, Hanna Siiskonen, Anita Naukkarinen, Ilkka T. Harvima

**Affiliations:** ^1^Department of Dermatology, University of Eastern Finland and Kuopio University Hospital, 70210 Kuopio, Finland; ^2^Department of Pathology, University of Eastern Finland, 70210 Kuopio, Finland

## Abstract

Mast cells contribute to the pathogenesis of cutaneous vasculitis through complement C3 that is cleaved to C3b and then to iC3b by complement factor I. The receptor of iC3b, CD11b, is expressed on neutrophils and monocytes and CD14 on monocytes. Their role in vasculitis is obscure. In this study, frozen skin biopsies from the nonlesional skin, initial petechial lesion, and palpable purpura lesion from 10 patients with immunocomplex-mediated small vessel vasculitis were studied immunohistochemically for complement factor I, iC3b, CD11b, and CD14. Peripheral blood mononuclear cells from 5 healthy subjects were used to study cell migration and cytokine secretion. Already, the nonlesional skin revealed marked immunostaining of complement factor I, iC3b, CD11b, and CD14, and their expression increased sequentially in initial petechial and palpable purpura lesions. Mast cell C3c correlated to iC3b, and both of them correlated to CD11b^+^ and CD14^+^ cells, in the nonlesional skin. The stimulation of mononuclear cells with 0.01-0.1 *μ*g/ml iC3b induced cell migration in the transwell assay. C3a stimulated slightly interleukin-8 secretion, whereas 1 *μ*g/ml iC3b inhibited it slightly, in 4/5 subjects. In conclusion, the C3-C3b-iC3b axis is activated already in the early vasculitis lesion leading to progressive accumulation of CD11b^+^ and CD14^+^ cells.

## 1. Introduction

Immunocomplex-mediated cutaneous small vessel vasculitis affects postcapillary venules and is characterized by neutrophil accumulation, leukocytoclasis, and fibrinoid necrosis in the vessel wall. After precipitation of immunocomplexes onto vessel walls, the complement system is activated resulting in vessel damage. These events can be seen in direct immunofluorescence (IF) staining of an early vasculitis lesion as positive vessel wall staining of immunoglobulins; complement products, including C1q and C3; and fibrin [1, 2].

Mast cells and their Fc*γ* receptors contribute to the immunocomplex-mediated vasculitis in animal models [3–5]. However, the knowledge regarding mast cells in human vasculitis is sparse. Previously, the serine proteinases of mast cells, tryptase and especially chymase, were found to correlate to the IF staining for C3c and fibrin in the initial petechial (IP) and/or palpable purpura (PP) lesions of immunocomplex-mediated cutaneous vasculitis [6]. After the discovery by Fukuoka et al. that isolated human skin mast cells express and constitutively secrete complement C3 *in vitro* [7], it was found that mast cells in cutaneous vasculitis contain C3c immunoreactivity. Further, C3 and C3a were subject to degradation by recombinant human chymase [8]. In fact, cutaneous mast cells contain C3c immunoreactivity in psoriasis and keratinocyte skin cancers, and the proportion of C3c^+^ mast cells correlates positively to CD11b^+^ cells in both the nonlesional and the lesional skin [9].

Upon activation of the complement system, C3 is converted to C3a and C3b. Thereafter, C3b is cleaved and inactivated to iC3b in a two-site cleavage by complement factor I (CFI), in cooperation with cofactors. The products, C3b and iC3b, remain covalently attached to C3b-acceptor sites, e.g., on the cell membrane. Even though iC3b represents an inactivated C3b product, it is immunologically active, as it is the ligand of the complement receptor CR3 (CD11b/CD18) [10]. During the cleavage of C3b to iC3b, a novel three-dimensional structure is formed [11]. CD11b/CD18 has been suggested to play a proinflammatory role in a variety of diseases. Nevertheless, this integrin receptor can mediate immunosuppressive actions, too [12]. In agreement with this multifaceted role of CD11b/CD18, CD11b is expressed on neutrophils, monocytes, and myeloid-derived suppressor cells (MDSC) [12, 13]. CD11b can work in physical or functional collaboration with CD14 that is a pattern-recognition receptor expressed by monocytes and macrophages [12, 14].

The purpose of this work was to study CD11b^+^ and CD14^+^ cells and their relation to C3c, iC3b, or CFI immunoreactivity during the progress of immunocomplex-mediated cutaneous small vessel vasculitis. Therefore, frozen skin biopsies from the nonlesional skin and IP and PP lesions of 10 patients with cutaneous vasculitis were examined using immunohistochemical techniques. In addition, the cell numbers were correlated to the direct IF staining results. Peripheral blood mononuclear cells (PBMCs) were isolated from 5 healthy donors to investigate the effect of purified human iC3b on cell migration and cytokine production.

## 2. Materials and Methods

### 2.1. Patients

The patients with immunocomplex-mediated small vessel vasculitis on the skin of lower limbs (4 females and 6 males, aged 56-86 years) have been described previously [6]. Briefly, 4 mm punch biopsies were taken from the (1) nonlesional skin, (2) IP lesion, and (3) PP lesion. The biopsies were taken before any effective systemic or local treatment. After removal, 5 *μ*m thick cryosections were prepared. The presence of immunoreactants in vessel walls was confirmed in direct IF staining by a well-experienced medical cell biologist (AN) using antibodies against IgM, IgA, IgG, fibrinogen, and C3c [6]. The protocol used was approved by the Ethics Committee of Kuopio University Hospital, Kuopio, Finland. The patients gave their informed consent prior to inclusion in the study.

### 2.2. Immunohistochemical Staining of CD11b, CD14, iC3b, and Complement Factor I

The immunohistochemical stainings of CD11b, CD14, and iC3b have been described in our recent study [9]. CD11b was immunostained using 0.5 *μ*g/ml mouse monoclonal anti-CD11b antibody (clone C11b/660) purchased from Novus Biologicals (Abingdon, U.K.), and CD14 was stained using 0.3 *μ*g/ml mouse monoclonal anti-CD14 antibody (clone TÜK4) from Dako Denmark A/S. The complement iC3b was stained using 0.06 *μ*g/ml mouse monoclonal antihuman iC3b (neo) antibody (catalog number A209, IgG2b*κ*, Quidel® Corporation, San Diego, CA, USA). CFI was stained using 20 *μ*g/ml mouse monoclonal antihuman CFI antibody (catalog number MCA2615, Bio-Rad, Oxon, UK). The stainings were controlled using unrelated mouse IgG. The primary antibody was visualized using the Vectastain Elite ABC kit (Vector Laboratories, Burlingame, CA, USA). Three cryosections per slide were analysed. The staining intensity of iC3b in the upper dermis was analysed using semiquantitative scoring: “1” = weak staining, “2” = moderate staining, and “3” = strong staining. CD11b^+^, CD14^+^, and CFI^+^ cells were counted beneath the epidermis using a 40x objective and ocular grid. The results are expressed as cells/mm^2^.

In addition, the previous results on C3c immunoreactivity in cutaneous mast cells in vasculitis were used in this study. The polyclonal antibody against C3c reacts both with C3c and with the C3c part of native C3 and C3b [8].

### 2.3. Sequential Immunohistochemical Staining of Complement Factor I and Mast Cell Tryptase

The technique has been described previously [9, 15]. Shortly, after immunohistochemical staining of CFI, all cryosections were photographed using a 20x objective. Thereafter, the cryosections were stained immunohistochemically using 0.1 *μ*g/ml rabbit polyclonal antitryptase antibody [16] and Vectastain ABC-AP kit (alkaline phosphatase, rabbit IgG, with biotinylated goat antirabbit IgG). The stainings were controlled using mouse or rabbit IgG. The tryptase-stained sections were rephotographed at exactly the same sites as previous pictures.

### 2.4. Isolation and Stimulation of Peripheral Blood Mononuclear Cells

A heparinized peripheral blood sample was drawn from the antecubital vein of 5 healthy volunteers (4 females and 1 male, aged 24-39 years). PBMCs were separated by Ficoll-Paque™ PLUS (GE Healthcare Bio-Sciences, Uppsala, Sweden) at 1000 × g for 10 min [17]. The PBMCs were collected from healthy subjects assuming that they mimic reasonably well the cells of patients with a very early vasculitic event in the nonlesional dermal skin (see Results).

PBMCs (1 × 10^6^ cells per ml of RPMI-1640 medium without serum) were added to wells of a 96-well plate followed by addition of the stimulant proteins, 5 or 25 *μ*g/ml complement C3a (final concentration) (R&D Systems Europe, Oxon, UK) or 1, 5, or 25 *μ*g/ml purified human iC3b (catalogue number 204863, Merck, Darmstadt, Germany) or diluent control in triplicate wells for incubating at 5% CO_2_ and +37°C for 24 hours. After incubation, a sample taken from each well was frozen for the subsequent analysis of interleukin- (IL-) 1*α*, IL-2, IL-6, IL-8, IL-10, IL-12, IL-17 and granulocyte-macrophage colony stimulating factor (GM-CSF) using Human Common Cytokines Multi-Analyte ELISArray™ kit (MEH-006A) (Qiagen, Nordic, Helsinki, Finland).

In the cell migration experiment with PBMCs from these same 5 healthy subjects, BD Biocoat™ Matrigel™ control chambers (BD Biosciences Europe, Erembodegem, Belgium) for 24-well plates were used. Purified human iC3b was diluted in 1% heat-inactivated bovine serum albumin in phosphate-buffered saline, and 0.01, 0.1, 1, 5, or 25 *μ*g/ml (final concentration) of iC3b or diluent control was added to duplicate wells. Thereafter, PBMCs (1 × 10^6^ cells per ml of RPMI-1640 medium with penicillin and streptomycin, but without serum) were added into the transwells. The media in lower and upper transwells were adjusted to the same level. The plates were incubated at 37°C and 5% CO_2_ for 48 hours. After incubation, the nonmigrating cells were rinsed and scrubbed out from the upper surface of the membrane using repeated washing with D-PBS. The attached cells on the lower surface of the membrane were fixed in cold methanol for 10 min and stained with May-Grünwald-Giemsa stain. After staining, the membranes were detached with a scalpel and transferred onto glass slides using Entellan® new rapid mounting medium (Merck, Darmstadt, Germany). The cells were counted in 9 randomly chosen high power fields (HPF) using a 20x objective and ocular grid. The cells were counted in a blinded fashion. For the identification and interpretation of migrated cell lineages, a hematologist at Kuopio University Hospital was consulted.

### 2.5. Statistical Analysis

The difference in the number of CD11b^+^, CD14^+^, and CFI^+^ cells was analysed using a two-tailed paired *t*-test. The difference in the staining score of iC3b was tested using mixed model analysis. The correlation between variables was tested using the Spearman correlation test. Values with *p* < 0.05 were considered significant.

## 3. Results

### 3.1. The Score of iC3b and the Number of CFI-Positive Cells

**Figure 1:**
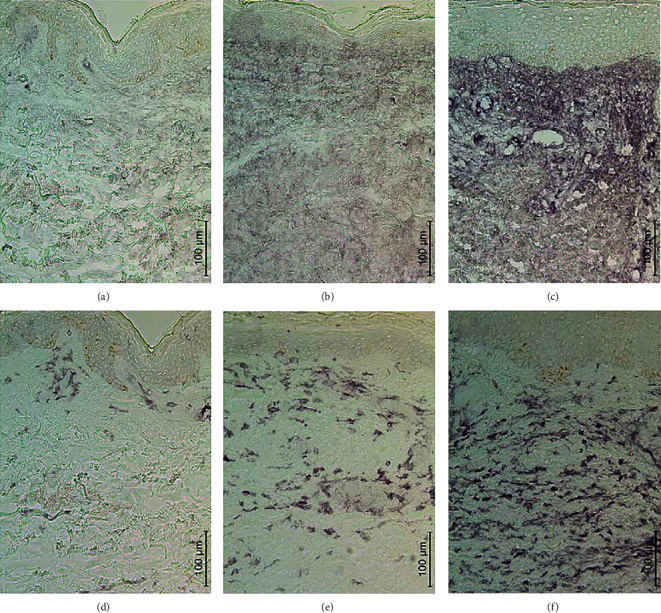
The immunohistochemical staining of iC3b (a–c) or CD11b (d–f) in the nonlesional skin (a, d), initial petechial lesion (b, e), and palpable purpura lesion (c, f) of a patient with vasculitis. The micrographs were taken using a 20x objective (scale bar 100 *μ*m).

The score of iC3b increased significantly in the PP lesion compared to the nonlesional skin (Table 1 and Figure 1). Marked numbers of CFI^+^ cells were observed already in the nonlesional skin and significantly increased numbers in the IP lesion, but no further rise was observed in the PP lesion (Table 1), though there was high variation in CFI^+^ cells between 10 subjects. Thus, iC3b increased later than CFI.

### 3.2. The Number of CD11b- and CD14-Positive Cells

The numbers of CD11b^+^ cells were significantly higher in IP and PP lesions than in the nonlesional skin (Table 1 and Figure 1). Likewise, the numbers of CD14^+^ cells were significantly increased in IP and PP lesions (Table 1; for a representative figure of the staining, see reference [9]). The correlation between CD11b^+^ and CD14^+^ cells was significant in the nonlesional skin (*r* = 0.733, *p* = 0.016), IP lesion (*r* = 0.915, *p* < 0.001), and PP lesion (*r* = 0.714, *p* = 0.047).

### 3.3. The iC3b Score and C3c-Positive Mast Cells and Their Correlation to CD11b- and CD14-Positive Cells

**Figure 2:**
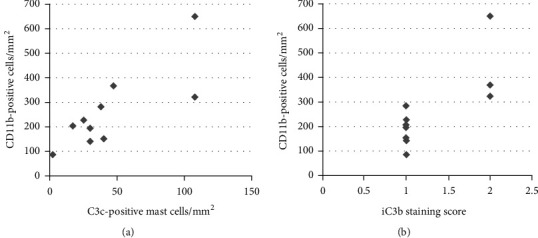
The correlation of (a) the number of C3c-positive mast cells (*r* = 0.726, *p* = 0.018) or (b) the staining score of iC3b (*r* = 0.798, *p* = 0.006) to the number of CD11b-positive cells in the nonlesional skin of 10 patients with vasculitis.

The score of iC3b correlated significantly to CD11b^+^ (*r* = 0.798, *p* = 0.006) (Figure 2) and CD14^+^ (*r* = 0.722, *p* = 0.018) cells in the nonlesional skin. No such correlations were observed in IP and PP lesions.

The percentage and number of mast cells containing C3c immunoreactivity (the values were from the previous study [8]) were correlated to CD11b^+^ and CD14^+^ cells in each skin sample group. The number of C3c^+^ mast cells correlated to CD11b^+^ cells in the nonlesional skin (*r* = 0.726, *p* = 0.018) (Figure 2), but the percentage of C3c^+^ mast cells did not (*r* = 0.527, *p* = 0.117). Instead, the percentage of C3c^+^ mast cells correlated to CD14^+^ cells in the nonlesional skin (*r* = 0.661, *p* = 0.038), but not the number of C3c^+^ mast cells (*r* = 0.591, *p* = 0.072). No such correlations were seen in IP and PP lesions.

The number, but not the percentage, of C3c^+^ mast cells correlated to the iC3b score in the nonlesional skin (*r* = 0.803, *p* = 0.005). This correlation was also significant in PP lesions (*r* = 0.764, *p* = 0.027), but not in IP lesions (*r* = 0.603, *p* = 0.065).

### 3.4. Complement Factor I and Tryptase-Positive Mast Cells

**Figure 3:**
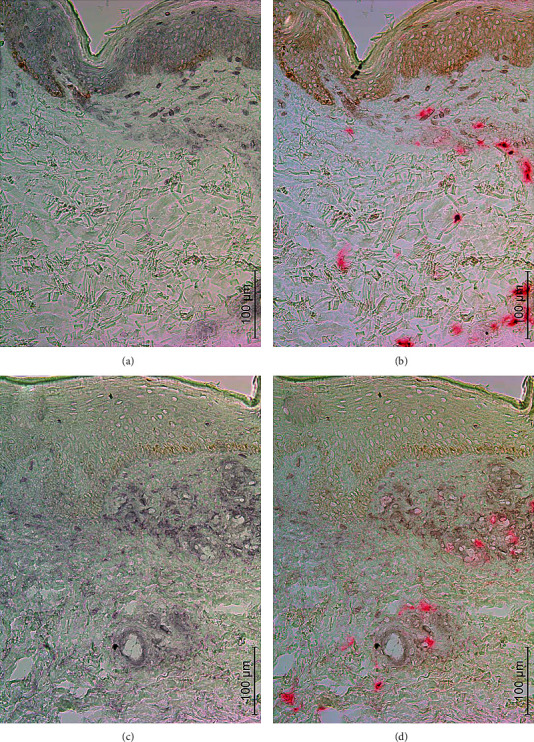
A sequential immunohistochemical staining of first complement factor I (a, c) and then mast cell tryptase (b, d). The skin biopsies were from the nonlesional skin (a, b) and palpable purpura lesion (c, d) of a patient with vasculitis. The micrographs were taken using a 20x objective (scale bar 100 *μ*m).

No apparent cytoplasmic CFI immunostaining in tryptase^+^ cells was detected in any of the nonlesional and lesional skin samples (Figure 3). The slight background-like CFI staining appeared to increase to a varying extent in the upper dermis during the progress of vasculitis. Interestingly, in many cases, tryptase^+^ cells were in apparent contact with CFI^+^ cells or background-like staining (Figure 3).

### 3.5. Mast Cell C3c, iC3b, CD11b, or CD14 and the Direct Immunofluorescence Staining Score of Vessel Wall C3c

The direct IF staining scores of vessel wall C3c (the values were from the previous work [6]) were correlated to the iC3b score, to CD11b^+^ and CD14^+^ cells, and to the number and percentage of C3c^+^ mast cells in each skin sample group. However, no significant correlation was observed between any of these variables.

### 3.6. Purified Human iC3b Induced Migration of Peripheral Blood Mononuclear Cells

**Figure 4:**
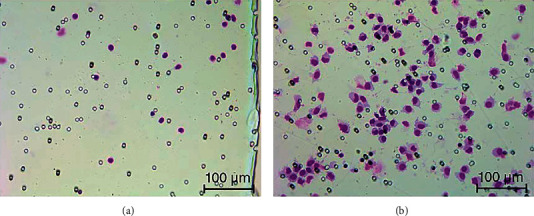
A representative micrograph showing migrated peripheral blood mononuclear cells (PBMCs) on the lower surface of porous membrane after stimulation for 48 hours with (a) diluent control or (b) 25 *μ*g/ml iC3b. The PBMCs, stained with May-Grünwald-Giemsa, were from the subject F29. The cells in Figure 4(b) belong mostly to the monocytic lineage, though they cannot be distinguished with absolute certainty from lymphocytes.

In the experiment with PBMCs from 5 healthy subjects, the mean ± SD number of attached white blood cells on the lower surface of porous membranes was 4.5 ± 2.3, 5.6 ± 1.8, 13.4 ± 11.1, 7.1 ± 3.7, 9.5 ± 14.9, and 24.8 ± 43.2 in HPF at 0, 0.01, 0.1, 1, 5, and 25 *μ*g/ml iC3b, respectively. The cells belong mostly to monocytic and/or lymphocytic lineages. All subjects showed an increase in cell number at either 0.01 (2 subjects) or 0.1 (3 subjects) *μ*g/ml iC3b. However, 2 subjects showed reversal to decreased cell numbers after initial peaking, but 3 subjects showed an increased cell number even at the highest iC3b concentrations, resulting in high variation in cell numbers. A representative micrograph is shown in Figure 4. Only occasional granulocytic cells were identified, and they did not show any increase in cell number.

### 3.7. Purified Human iC3b or C3a and Cytokines in the Conditioned Medium of Peripheral Blood Mononuclear Cells

**Figure 5:**
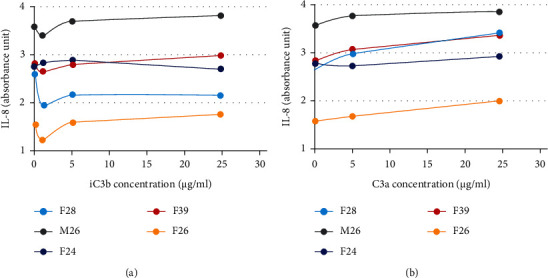
Stimulation of peripheral blood mononuclear cells from 5 healthy subjects for 24 hours using (a) iC3b and (b) C3a, and the resulting level of IL-8 in the conditioned medium in relation to the IL-8 level induced by the diluent control.

In all 5 subjects, already the baseline IL-8 level was high in the conditioned medium of PBMCs (Figure 5). In 4/5 subjects, iC3b at 1 *μ*g/ml reduced slightly this baseline level of IL-8 (Figure 5(a)). At higher iC3b concentrations, no further reduction in IL-8 levels was observed, but rather, it was reversed back or close to the baseline. In one subject, only the highest iC3b concentration reduced slightly the IL-8 level. By comparison, C3a increased slightly in a dose-dependent fashion the IL-8 level in 4/5 subjects (Figure 5(b)).

In the case of other cytokines, the changes were variable between subjects. Neither iC3b nor C3a induced any changes in the levels of IL-1*α* and IL-2. In one subject (F39), an increase in the IL-6 level was detected at 5 *μ*g/ml iC3b, and in another subject (F26), all concentrations of iC3b increased similarly the level of IL-12. In these 2 same subjects, the IL-10 level was increased at 1 *μ*g/ml, but not so at 5 and 25 *μ*g/ml iC3b, and in the case of F26, the IL-17 level was increased at 1-25 *μ*g/ml iC3b.

The measurement of GM-CSF produced also variable results in 3 subjects. In the case of F26, 1-25 *μ*g/ml iC3b reduced in a dose-dependent fashion the spontaneously slightly elevated level of GM-CSF as did also 5 and 25 *μ*g/ml C3a. In the case of F39, an elevated level of GM-CSF was measured at 5 *μ*g/ml iC3b and 25 *μ*g/ml C3a. In the case of F24, the level of GM-CSF was elevated at 25 *μ*g/ml C3a and 1 *μ*g/ml iC3b. The remaining 2 other subjects showed no changes in the GM-CSF level.

## 4. Discussion

Mast cells can contribute to the immunocomplex-mediated vasculitis [3–5], but their role in human vasculitis is unclear. Human mast cells have been shown to express the Fc*γ*RIIa receptor [18] as well as the high-affinity IgG receptor, Fc*γ*RI/CD64, after stimulation of *in vitro*-cultured mast cells by interferon-*γ* [19]. In addition, mast cells can be activated by C3a generated from C3 [20, 21].

Human skin mast cells can constitutively produce C3 as well as proteases that process C3 to C3a and C3b, including cathepsin G, granzyme B, and monomeric *β*-tryptase [7, 22–26]. Therefore, mast cells have most of the machinery to initiate and perpetuate the C3-mediated reaction in vasculitis once activated. This hypothesis is supported by the demonstration of C3c immunoreactivity in mast cells in vasculitis [8]. Furthermore, the partial inactivation of chymase in vasculitis allows survival of C3 [6]. However, CFI immunoreactivity was not detected in mast cells in any vasculitis specimen, but on several occasions, tryptase^+^ mast cells were found in apparently close apposition to other CFI^+^ cells than mast cells. This suggests that C3b produced from mast cell C3 is further cleaved to iC3b by CFI derived from another cell type. Furthermore, the immunostainings increased sequentially in a logic fashion; that is, CF1^+^ cells increased significantly first in the IP lesion followed by subsequent increase in the score of iC3b in the PP lesion. Because the number of C3c^+^ mast cells correlated to the score of iC3b in the nonlesional skin, these cells can be assumed to be one marked source for C3, C3b, and iC3b in the very early events of vasculitis. In the recent study, no marked cytoplasmic iC3b staining was detected in mast cells in the nonlesional or lesional skin samples from a variety of skin diseases, but in many instances, tryptase^+^ mast cells were observed in close apposition to iC3b immunoreactivity [9]. Furthermore, the prominent immunostaining of C3c in tryptase^+^ cells also suggests that mast cells are a marked source for the iC3b detected extracellularly around them [8, 9]. Nevertheless, the staining scores of vessel wall immunoreactants, including C3c, did not correlate to mast cell C3c, iC3b, or other immunomarkers suggesting that the source for vessel wall immunopositivity is the blood. In this context, it should be noted that the nonlesional skin of vasculitis does not represent normal skin, because weak vessel wall positivity of immunoreactants was detected in direct IF staining in these healthy-looking skin samples [6].

Previously, increased numbers of CD11a^+^, CD11b^+^, and CD11c^+^ cells have been counted in leukocytoclastic vasculitis lesions [27]. In this study, the increase in iC3b immunoreactivity in IP and PP lesions coincided with the accumulation of CD11b^+^ and CD14^+^ cells, and there was a significant correlation between CD11b^+^ and CD14^+^ cells in each lesion suggesting at least some colocalization of CD11b and CD14 to same cells, that is, plausible monocytes and macrophages [12, 14]. Without functional analyses of isolated cells, it is difficult to confirm whether there are CD11b^+^CD14^+^HLA-DR^−^/^low^CD15^−^ M-MDSC cells with immunosuppressive properties [13]. Especially in the nonlesional skin, the number of CD11b^+^ cells was close to that of CD14^+^ cells, but the rest of the predominating CD11b^+^ cells in IP and PP lesions can be attributed to neutrophils [12]. The number or percentage of C3c^+^ mast cells as well as the iC3b score correlated to CD11b^+^ and CD14^+^ cells in the nonlesional skin, which suggests a role for the C3-C3b-iC3b axis in attracting these plausible monocytic cells already in the very early, though clinically healthy-looking, lesion. The migration experiment with PBMCs supports this conclusion. Once the vasculitis process was clinically advanced in the IP followed by PP lesion, neutrophils and additional monocytic cells progressively accumulated from the blood circulation. It is unlikely that the CD11b^+^ cells are mast cells because CD11b has not been detected on human skin mast cells [28].

After UVB irradiation, the deposition of iC3b at the dermal-epidermal junction has previously been found to lead to the appearance of CD11b^+^ cells to the same site [29]. In the mouse skin model, C3 and its activation products are essential for UV-induced immunosuppression to a contact sensitizer and also for the accumulation of CD11b^+^ cells [30]. The activation of CD11b/CD18 on monocytes or antigen-presenting cells by iC3b can result in immunosuppression or immunotolerance through upregulation of IL-10 and TGF-*β* but downregulation of IL-12 [29, 31]. Further, the differentiation of monocyte-derived dendritic cells can be arrested by iC3b [32]. The pathogenetic role of CD11b^+^ and CD14^+^ cells in immunocomplex-mediated vasculitis is unclear. It is possible that the C3-C3b-iC3b axis derived from mast cells or keratinocytes in the nonlesional or normal skin has physiologic function in the innate immunity by opsonizing microbes, clearing immunocomplexes, and preventing excessive immune activation. However, the primary etiopathogenetic events in immunocomplex-mediated vasculitis typically take place in the peripheral blood. The complement activation in response to immunocomplexes on vascular endothelium with subsequent vessel damage can be excessive, and therefore, the normal regulatory mechanisms in the dermal skin are exceeded by the disease. To further clarify the role of iC3b, *in vitro* experiments were performed. These experiments with PBMCs revealed that in 4/5 subjects, low concentration (1 *μ*g/ml) of iC3b slightly reduced the IL-8 level in conditioned medium, but higher iC3b concentration did not. In 2/5 subjects, an increased IL-10 level was induced, but only at 1 *μ*g/ml iC3b. However, the proteolytic processing of C3 produces anaphylatoxin C3a, too, and it can affect C3aR^+^ and CD11b^+^ dendritic cells [33]. Because C3a increased slightly IL-8 levels in the conditioned medium in 4/5 subjects, it is possible that C3a can overcome the slight inhibitory effect induced by diluted iC3b. C3a has previously been shown to induce IL-8 secretion from endothelial cells [34] and polymorphonuclear leukocytes [35]. IL-8, in turn, is a well-known chemoattractant of neutrophils [36] that characteristically accumulate in leukocytoclastic vasculitis.

In summary, the present study together with the previous one [8] suggests that mast cells are a marked source for C3 and C3b that is further cleaved by accumulating CFI^+^ cells to iC3b in the early vasculitis lesion. Already, the clinically uninvolved skin revealed marked immunostaining of CFI, iC3b, CD11b, and CD14. The iC3b protein can attract plausible monocytic CD11b^+^ and CD14^+^ cells to the site of evolving early inflammation through migration. C3a generated in parallel with C3b can further stimulate the accumulation of CD11b^+^ neutrophils, e.g., through IL-8. To target therapeutically these early mechanisms, the C3-C3b-iC3b axis may be a promising possibility. However, it is not truly known yet whether these early events represent a failed immunosuppressive attempt to take control over vasculitic inflammation or whether they constitute one essential proinflammatory event.

## Figures and Tables

**Table 1:** The number of CD11b^+^, CD14^+^, or complement factor I (CFI)^+^ cells and the staining score of iC3b in skin biopsies from patients with immunocomplex-mediated cutaneous small vessel vasculitis.

	CD11b (cells/mm^2^)	CD14 (cells/mm^2^)	CFI (cells/mm^2^)	iC3b (score)
Nonlesional skin (*n* = 10)	264 ± 162	224 ± 117	124 ± 144	1.3 ± 0.5
Initial petechiae (*n* = 10)	853 ± 605^∗^	576 ± 330^#^	270 ± 168^&^	1.6 ± 0.7
Palpable purpura (*n* = 8)	2040 ± 1202^∗∗^	1115 ± 606^##^	219 ± 69 (*n* = 9)	2.6 ± 0.7^∗∗∗^

The results are expressed as the mean ± standard deviation. “∗” denotes *p* = 0.014 and “∗∗” *p* = 0.004 when the CD11b^+^ cell numbers in the initial petechial lesion and palpable purpura, respectively, were compared to the corresponding cell numbers in the nonlesional skin (paired *t*-test); “#” denotes *p* = 0.008 and “##” *p* = 0.0024 when the CD14^+^ cell numbers in the initial petechial lesion and palpable purpura, respectively, were compared to the corresponding cell numbers in the nonlesional skin (paired *t*-test); “&” denotes *p* = 0.0169 when the CFI^+^ cell numbers in the initial petechial lesion were compared to the corresponding cell numbers in the nonlesional skin (paired *t*-test); and “∗∗∗” denotes *p* = 0.003 when the iC3b scores in the palpable purpura were compared to the corresponding scores in the nonlesional skin (mixed model analysis).

## Data Availability

The data may be available upon request through the corresponding author.

## Abbreviations

CFI: Complement factor I
IF: Immunofluorescence
IP lesion: Initial petechial lesion
PBMC: Peripheral blood mononuclear cell
PP lesion: Palpable purpura lesion.
